# Stenting Versus Endoscopic Vacuum Therapy for Anastomotic Leakage After Esophago-Gastric Surgery

**DOI:** 10.3390/jcm14197075

**Published:** 2025-10-07

**Authors:** Carlo Galdino Riva, Stefano Siboni, Matteo Capuzzo, Francesca Senzani, Lorenzo Cusmai, Daniele Bernardi, Pamela Milito, Andrea Lovece, Eleonora Vico, Marco Sozzi, Emanuele Luigi Giuseppe Asti

**Affiliations:** Division of General and Emergency Surgery, IRCCS Policlinico San Donato, University of Milan, 20097 Milan, Italy; carlettogaldino@gmail.com (C.G.R.); matteo.capuzzo.95@gmail.com (M.C.); frasenzani@gmail.com (F.S.); lorenzo.cusmai@gmail.com (L.C.); marcosozzi92@gmail.com (M.S.); emanuele.asti@grupposandonato.it (E.L.G.A.)

**Keywords:** gastric cancer, esophageal cancer, anastomotic leak, endoscopic vacuum therapy, self-expandable metal stents

## Abstract

**Background:** Anastomotic leakage (AL) is a major complication after esophago-gastric surgery, with incidence rates of 11–21% and mortality up to 14%. Early intervention is essential to reduce morbidity. Endoscopic treatments have advanced, with self-expandable metal stents (SEMSs) as the traditional standard (success ~90%), but they carry risks like migration, stenosis, and need for drainage. Endoscopic vacuum therapy (EVT), applying negative pressure to drain secretions and promote healing, has shown success rates of 66–100%. Limited comparative data exists from small retrospective studies. This study compares SEMS and EVT for safety and efficacy in AL management. **Methods:** A retrospective case–control study from a prospective database at our institution was performed (March 2012–2025). We included patients with AL post-esophageal/gastric surgery treated endoscopically (SEMS or EVT). We excluded patients treated with conservative or surgical management. Demographics, comorbidities, oncology, surgery type, leak details, treatments, and outcomes were collected. Primary outcome was complete healing of the leak, while secondary outcomes were time to success, number of procedures needed, hospital stay, complications, mortality. **Results:** From 592 resections, we extracted 68 AL (11.5%), 45 of which met the inclusion criteria (22 SEMS, 23 EVT). Groups were similar demographically, but SEMS had more respiratory issues (43% vs. 8.7%, *p* = 0.018). SEMS were used more after esophagectomy (86.4% vs. 56.5%, *p* = 0.004); EVT was performed mostly after gastrectomy (34.7% vs. 9.1%, *p* = 0.009). Success rate was 86.4% for SEMS vs. 95.6% for EVT (*p* = 1.000). Complications were significantly lower in EVT (8.3% vs. 50%, *p* = 0.001; SEMS: 36.4% migrations, 18.2% stenoses). Leak onset time, modality of diagnosis, and leak size were comparable among the groups. Need for jejunostomy was higher in EVT (43.5% vs. 9.1%, *p* = 0.015), while chest drains in SEMS (63.7% vs. 13.1%, *p* < 0.001). Hospital stays (33–38 days, *p* = 0.864) and mortality (22.7% vs. 8.7%, *p* = 0.225) were similar. No differences were observed in terms of long-term mortality (log-rank *p* = 0.815). **Conclusions:** SEMS and EVT are both effective for AL after esophago-gastric surgery. EVT offers fewer complications and shorter treatment, so it is favored especially for esophago-jejunal leaks.

## 1. Introduction

Anastomotic leak (AL) is one of the most feared complications after upper gastro-intestinal (UGI) surgery, with an incidence ranging from 11% to 21% and an associated mortality rate of up to 14% [1,2]. For this reason, an early diagnosis and a timely and effective treatment are of paramount importance to improve the associated outcome [3,4]. In the last two decades, endoscopic approaches have been demonstrated to be safe and effective, but the optimal treatment for AL remains controversial, even if several new techniques and devices have been recently developed [5,6].

Self-expandable metal stents (SEMSs) have been considered the gold standard of endoscopic treatment, with a success rate up to 90% [7]. SEMS cover the defect preventing extra-luminal spillage and allowing oral diet and a fast patient’s discharge. However, their use has raised some concerns, such as high rates of stent migrations, an increased risk of post-operative stenosis, and the need of external drainage to evacuate mediastinal or thoracic collections [6].

In the last decade, following the optimal results in wound healing [8] and colorectal surgery [9], endoscopic vacuum therapy (EVT) has emerged as an alternative endoscopic treatment for AL [10]. The continuous sub-atmospheric pressure applied to the anastomosis through a polyurethane sponge removes secretions and drains mediastinal collections, thus reducing bacterial proliferation, and stimulates mucosal perfusion favoring the granulation process [11]. In contrast to SEMS, EVT plays an active role in the healing of AL, with a reported success rate ranging from 66% to 100% [12].

Recent meta-analyses by Mandarino et al. [12] and Scognamiglio et al. [13] have compared these two techniques after UGI surgery, reporting higher leak closure rates and lower mortality with EVT. However, these studies are limited by the heterogeneous retrospective data, including variable definitions of AL, and the small sample size of retrospective studies included.

The aim of this study is to assess these gaps with a direct comparison of EVT and SEMS in a well-defined cohort from a single high-volume center, utilizing a standardized protocol for AL management after esophago-gastric surgery.

## 2. Materials and Methods

We performed a retrospective observational case–control study based on a prospectively collected database at the Department of General and Emergency Surgery of the IRCCS Policlinico San Donato (Italy).

All patients with AL following esophageal or gastric surgery for malignant and benign conditions that underwent an operative endoscopic treatment for the AL between March 2012 and March 2025 were identified and included. Malignant conditions included adenocarcinoma or squamous cell carcinoma of the esophagus and adenocarcinoma of the proximal stomach. Benign conditions included patients with end-stage achalasia after failure of Heller–Dor myotomy and rescue pneumatic dilation. All the patients underwent either a hybrid (laparoscopy and thoracotomy) or totally minimally invasive (laparoscopy and thoracoscopy) esophagectomy. Surgery was performed by an experienced physician. Patients treated with a conservative approach or surgical revision were excluded.

A review chart was performed to extract demographic characteristics, comorbidities, oncologic staging, type of surgery, leak treatment, endoscopic features, and outcomes.

In our clinical practice, if an AL was suspected, the patient underwent either a CT-scan with oral contrast or an upper-GI endoscopy (EGD) to confirm the hypothesis. If the AL was confirmed, an expert endoscopist performed an EGD in the operating room under general anesthesia. In case of total disruption of the anastomosis or necrosis of the gastric conduit, a surgical revision was performed. Patients with small defects (<3 mm), well-vascularized mucosa, no extra-luminal collections, and without systemic involvement were managed conservatively with antibiotics and enteral nutrition via nasojejunal tube. All the other conditions underwent operative endoscopic procedures (SEMS or EVT) within 12 h from the diagnosis. The patients underwent SEMS or EVT based on physician preference.

### 2.1. Self-Expandable Metal Stent

After the endoscopic identification of the AL, SEMS was placed over a guidewire under fluoroscopic guidance and deployed to cover the leak. Either a fully covered (Ultraflex™ Esophageal NG Stent System, Boston Scientific, Marlborough, MA, USA) or partially covered (Niti-STM Esophageal Covered Stent, TaeWoong Medical Co., Gojeong-ro, Republic of Korea) SEMS were used. The type of stent was chosen by the endoscopist based on personal preference.

Every patient underwent an esophagogram with oral Gastrografin on post-procedure day 1 to ensure the correct stent placement. In case of successful procedures and good patient conditions, after the end of antibiotics treatment they were discharged on a semi-liquid diet and scheduled for an outpatient EGD after three weeks. If the continuity of the esophageal wall was restored after removal, the treatment was considered successful; otherwise, a new stent was placed.

### 2.2. Endoscopic Vacuum Therapy

EVT was introduced in our clinical practice in 2018. The principles behind this technique overlap those of the vacuum-assisted closure therapy used to treat complex wound defects [10]. Self-made EVT technique was used, as described elsewhere [14]. The patient underwent general anesthesia to safely manage the airways during the passage of the device.

**Figure 1:**
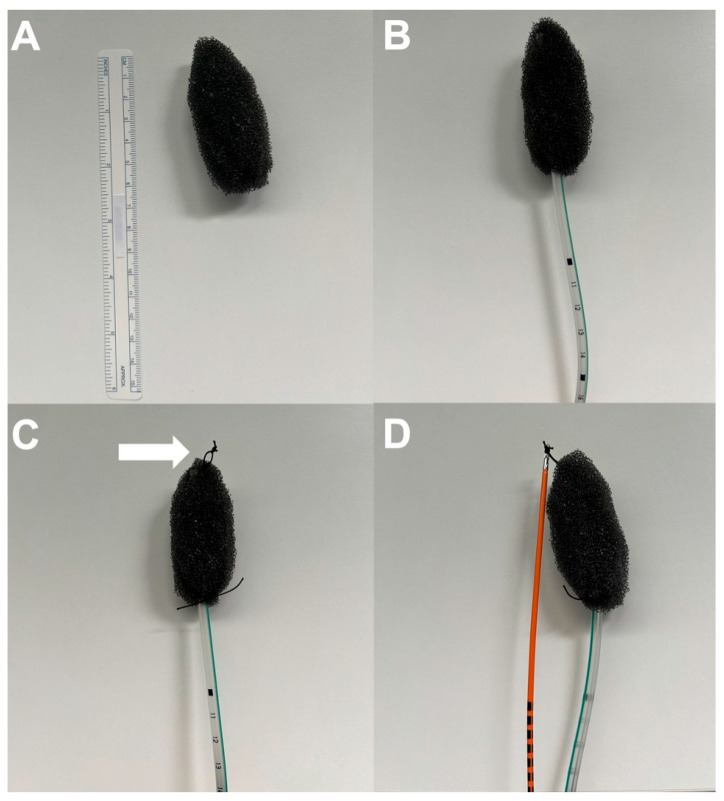
EVT preparation. (**A**) A standard sponge is shaped to fit in the defect anatomy. (**B**) The NG tube is passed through a tunnel created within the sponge. (**C**) The sponge is sewed to the tube with a proximal and distal silk suture; the distal one presented a loop (arrow) in order to facilitate its positioning. (**D**) The biopsy forceps grasps the loop to convey the device into the esophagus.

Following the initial endoscopic assessment of the fistula, which allowed precise measurement of the defect, a silicon nasogastric (NG) tube was inserted through one nostril and retrieved from the patient’s mouth. An open-cell foam (VAC—GranuFoam) was shaped from a standard sponge to fit the defect anatomy (Figure 1A). A tunnel was created within the tailored EVT to allow the passage of the NG tube (Figure 1B). The EVT was then secured to the end of the NG tube using proximal and distal silk sutures, with a loop left at the distal end to aid insertion with biopsy forceps (Figure 1C). All fenestrations of the NG tube were positioned within the foam to optimize suction. The sponge was then conveyed into the esophagus by the biopsy forceps (Figure 1D) inserted into the operative channel of the endoscope and placed either intracavitary (for defects larger than the endoscope with a mediastinal cavity) or intraluminal (for smaller defects). Once positioned, the NG tube was connected to a suction pump set to a continuous negative pressure of −125 mmHg. The procedure was repeated every 72–96 h until the leak was sealed.

A hybrid procedure involving the placement of a SEMS was performed in two scenarios: First, the SEMS was placed over the EVT when the EVT alone failed to achieve complete sealing of the leak; second, when the defect was nearly closed and no cavity was observed during endoscopy.

During the EVT treatment, patients were hospitalized and fed either by parenteral nutrition (PN) via central venous catheter or enteral nutrition (EN) via jejunostomy, surgically placed during the first endoscopic procedure. The choice of PN vs. EN was based on the leak dimension and the nutritional status of the patient.

### 2.3. Outcomes and Statistical Analysis

The primary outcome was the clinical success rate, defined as endoscopic evidence of complete healing of the leakage. Clinical failure was defined as persistent leakage during follow-up, need for surgical resection due to persistent leakage, or death before treatment completion. Secondary outcomes included time to achieve clinical success, number of endoscopic procedures required for success, length of the hospital stay, complication rate, and hospital mortality.

Categorical variables are presented as frequency and percentages while continuous variables as median and interquartile range (IQR). Categorical variables were compared using the chi-squared test or the Fisher exact test as appropriate. The normality of continuous variables was assessed with the Shapiro–Wilk test. Normal variables were compared with the *t*-test and nonparametric variables using the Kruskal–Wallis test.

Survival analysis was performed using the Kaplan–Meier method to estimate overall survival probabilities from surgery to the time of death. Differences between treatment groups were assessed using the log-rank test, and hazard ratios were calculated using Cox proportional hazards regression.

A *p*-value of less than 0.05 was considered statistically significant.

Statistical analysis was conducted using R software version 4.4.2 (The R Foundation for Statistical Computing, Vienna, Austria).

## 3. Results

**Figure 2:**
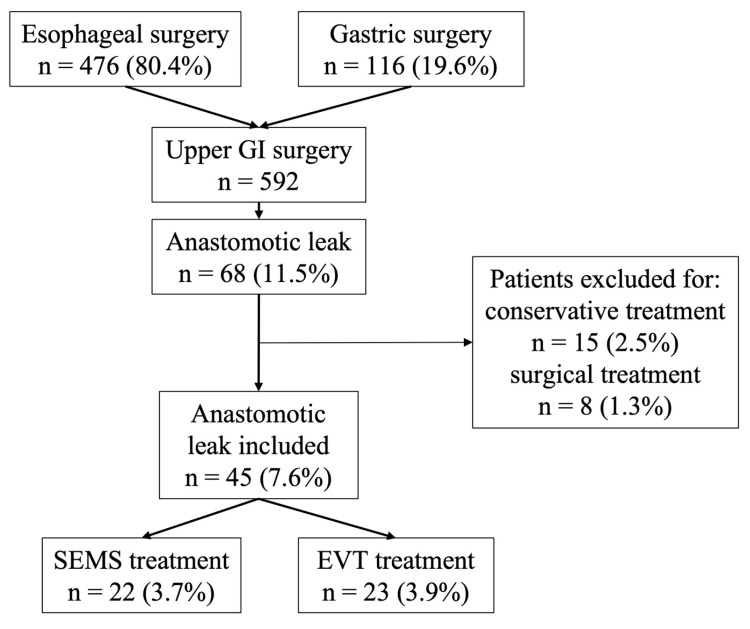
Flow chart of the cohort of patients included in the study. GI: Gastrointestinal; SEMS: Self-expanding metal stent; EVT: Endoscopic vacuum therapy.

Between March 2012 and March 2025, a total of 592 patients underwent esophageal (n = 476) or gastric (n = 116) resections. A total of 68 patients (11.5%) developed an anastomotic leak, 55 (11.5%) after esophageal and 13 (11.2%) after gastric resection. After the exclusion of 23 patients that did not meet the inclusion criteria, 45 patients were enrolled, 22 treated with SEMS (48.9%) and 23 with EVT (51.1%). The flow chart of the study is reported in Figure 2.

The two groups were comparable in terms of demographic and clinical characteristics, with only a significantly higher rate of respiratory comorbidities in the SEMS group compared to EVT (43% vs. 8.7%, *p* = 0.018) (Table 1). Oncologic features, such as staging, histotype, and neoadjuvant therapy were similar between the groups, in patients with both esophageal and gastric cancer. We found significant differences in terms of surgical technique, with SEMS being more commonly used after Ivor–Lewis esophagectomy (86.4% vs. 56.5%, *p* = 0.004), while EVT predominantly used after total gastrectomy with abdominal esophago-jejunal anastomosis (34.7% vs. 9.1%, *p* = 0.009).

**Figure 3:**
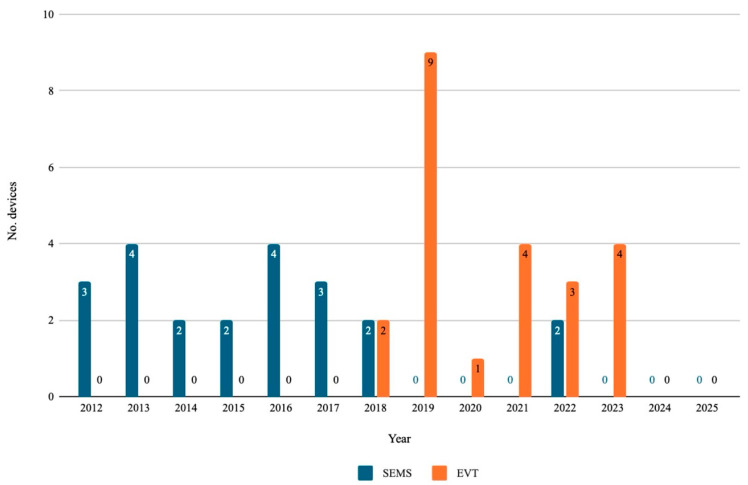
Trend of anastomotic leaks endoscopic treatment between 2012 and 2025. SEMS: Self-expanding metal stent; EVT: Endoscopic vacuum therapy.

The trend of endoscopic procedure type performed over the time span of the study is shown in Figure 3.

As shown in Table 2, there was no difference in terms of time interval between surgery and AL diagnosis (*p* = 0.882), nor in terms of modality of the diagnosis (EGD vs. CT-scan, *p* = 0.897) or defect size (*p* = 0.135). Eight patients (34.8%) in the EVT group started the treatment with an intracavitary sponge, while in all other cases the defect was treated positioning the sponge directly intraluminal.

A significantly lower median number of devices was used to treat the leak in the SEMS group (2.0 vs. 5.0, *p* < 0.001), but the median treatment duration was shorter in the EVT group (22 vs. 32 days, *p* = 0.029). Additional procedures included surgical jejunostomy in 43.5% of the EVT group and 9.1% in the SEMS group (*p* = 0.015). Chest-drain placement was significantly higher in the SEMS group compared to the EVT group (63.7% vs. 13.1%, *p* < 0.001). In the EVT group, six patients (26.1%) required stent placement during the initial phase of the treatment to fully exclude the leak or toward the end to facilitate an oral diet.

Table 3 shows no significant differences in clinical success rate (86.4% in the SEMS group vs. 95.6% in the EVT, *p* = 1.000), while significant differences emerged in the total number of adverse events (50% SEMS vs. 8.3% EVT, *p* = 0.001). No independent predictors of clinical success have been found when included in a multivariable model (Supplemental Table S1). Among the 12 adverse events in the SEMS group, eight were migrations and four short-term stenosis. No statistically significant differences were observed in overall length of hospital stay (*p* = 0.855) or hospital mortality (*p* = 0.225). Supplemental Figure S1 represents a schematic comparing SEMS vs. EVT workflows.

**Figure 4:**
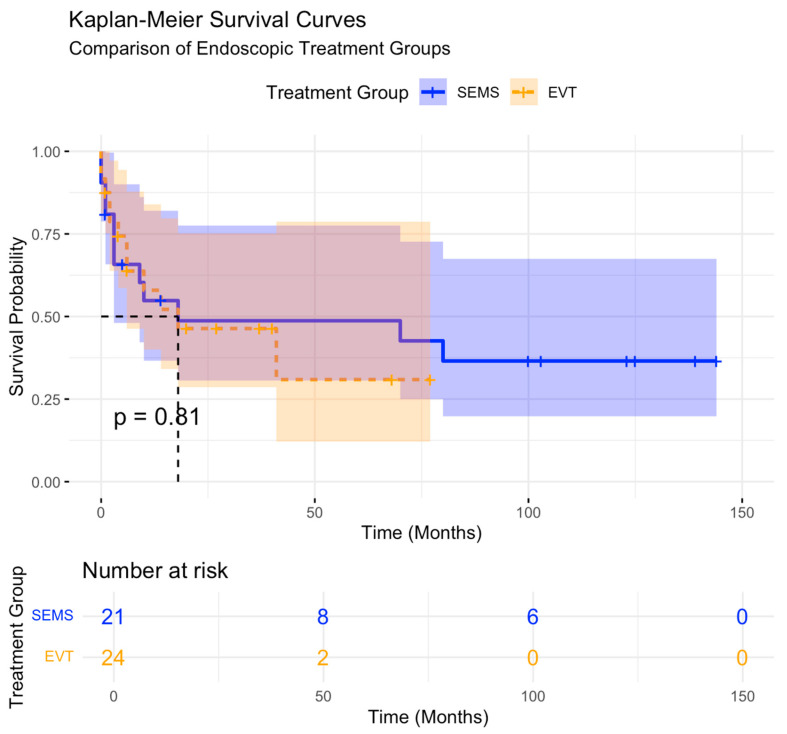
Kaplan–Mayer curve of overall survival in patients treated with endoscopic procedure for anastomotic leak. SEMS: Self-expanding metal stent; EVT: Endoscopic vacuum therapy.

Median overall survival was 18 months both in the EVT and in the SEMS groups. Kaplan–Meier survival analysis showed no statistically significant difference in overall survival between the groups (log-rank *p* = 0.815). Cox regression analysis confirmed no significant association between endoscopic treatment and mortality risk (Hazard Ratio = 1.09, 95% CI: 0.47–2.53, *p* = 0.832) (Figure 4). An adjusted Cox model integrating demographic and oncologic characteristics (i.e., age, stage, cancer site, anastomosis type and leak severity) was not feasible given the small number of patients in our study.

## 4. Discussion

Our study demonstrates that the clinical efficacy of EVT in the treatment of AL after upper-GI surgery is comparable to that of SEMS.

The management of AL has always been challenging for esophageal surgeons, given its variability in terms of clinical manifestation, severity and outcome. Moreover, its contribution to high post-operative morbidity, mortality, and oncological survival makes this topic of paramount importance [15]. The treatment of AL is not standardized and endoscopic procedures, such as SEMS and EVT, have been introduced in recent years to fill out the gap between a conservative approach and a surgical intervention, with good results [16].

An increased number of retrospective studies with a small cohort of patients described these two techniques [12,13]. The main advantage of the EVT is its active role in promoting leak healing and continuously suctioning extra-luminal collection. However, its limitations include the inability to maintain an oral diet and the potential for delayed patient discharge.

Our study clearly demonstrates these features, showing that patients in the EVT group had a significantly lower rate of chest drain (13.1% vs. 63.7%, *p* < 0.001) but a significantly higher rate of jejunostomy (43.5% vs. 9.1%, *p* = 0.015). The significantly lower rate of chest drain in EVT group should be related also to the fact that most patients treated with EVT suffered from a leak of an esophago-jejunal anastomosis, with only abdominal contamination.

Moreover, given the critical role of enteral nutrition in these patients, we opted to provide enteral access for those expected to require prolonged treatment. This is of paramount importance in these patients since a good nutritional status plays a crucial role in wound healing.

In our cohort, SEMS was more frequently used in AL after esophagectomy, while EVT after gastrectomy, as reported in past studies [17,18,19]. Patients with esophago-jejunal anastomosis pose significant challenges for SEMS treatment, due to the anastomosis asymmetry [20], the presence of the cul-de-sac that does not allow a complete exclusion of the leak and a frequent stent migration in up to 61% of cases [21]. For these reasons and based on our data, we think that the treatment of choice in these patients should be EVT.

EVT and SEMS were both safe and effective procedures. Despite their use in urgent settings, less than 30% of patients experienced minor complications, with no major adverse events reported in our series. Terceiro De Oliveira et al. reported similar findings in a cohort of patients treated with SEMS and EVT for traumatic esophageal rupture [22]. However, our analysis showed a significantly higher complication rate in the SEMS group, particularly due to stent migration. This may be attributed to the fact that the SEMS were mostly fully covered, and consequently more prone to this complication as also described in a recent retrospective work by Heilani et al. [23]. No bleeding episodes, typically associated with EVT, were observed in our series. While both treatments were comparable, larger and more robust studies are needed. The ongoing phase II randomized controlled trial (ESOLEAK trial; NCT03962244) comparing EVT and SEMS for anastomotic leakage after Ivor–Lewis esophagectomy is expected to provide stronger evidence [24].

The most recent systematic review, published in 2023 by Mandarino et al., included eight retrospective studies for a total of 357 patients. EVT treatment demonstrated better results in terms of success rate, number of devices used, treatment duration, short-term complication and mortality rate [12]. In our series, SEMS and EVT appeared to be both effective procedures with high rates of technical (100%) and clinical (>90%) success, probably due to the well-standardized protocol of AL management and the experienced endoscopy service in our third-level hospital. Also, we strongly believe that a timely diagnosis and treatment is the most important factor for AL treatment. Additionally, with our study we confirm the superiority of EVT over SEMS in terms of treatment duration and overall complications. Nevertheless, in our study the number of devices used was significantly higher in the EVT group, given the need for device change every 72–96 h to assess the AL healing and to tailor the sponge.

As also described by Chon et al. [25], six patients treated with EVT required the addition of a SEMS to achieve complete AL healing. In three cases, the SEMS was placed at the start of treatment, to allow complete sealing of the leak, while in the remaining three cases the SEMS was placed at the end of EVT treatment, when the AL gap had narrowed to speed up the sealing, allowing oral diet and patient discharge. We intended this hybrid approach as an optimization of both procedures to reduce the time of treatment.

In our study we observed a significantly higher number of endoscopic device changes in the EVT group, consistent with the existing literature [12,16]. This allowed more frequent endoscopic lavage and debridement during every change, reducing inflammation associated with AL [26,27] and accelerating healing. Consequently, the duration of EVT treatment was significantly shorter than that of SEMS, with a median of 22 days of treatment, as reported elsewhere [28], although the length of hospital stay was similar between groups (*p* = 0.855). Previously reported cost analysis has shown significantly higher costs for EVT, typically double those of SEMS [29,30]. However, our use of a hand-made device helped to narrow this gap, although a detailed cost analysis was not conducted in this study.

Our study has some limitations. First, its retrospective design introduces potential selection bias, which may influence outcomes, despite no significant demographic differences being observed. Second, the small patient cohort, consistent with most studies in the literature, and the lack of data needed for leak stratification (e.g., SEAL score) limit the power of our analysis. Third, variations in baseline conditions and surgical interventions may have impacted the results. The exclusion of patients treated with conservative or surgical approach may have introduced a selection bias, although the decision was driven by the aim of the study, which focused on endoscopic treatment. Finally, the data were biased by introduction of EVT into our clinical practice in 2018, with routine use for smaller AL only recently adopted. This limitation could have introduced a bias related to the physician learning curve and improvement in peri-operative care over time. Particularly, while SEMS was a well-established procedure, EVT required a longer learning curve to establish the correct modality of treatment (intra vs. extra-luminal, time between procedures). An era-adjusted analysis comparing outcomes before and after 2018 can be found on Supplemental Table S2.

Future research should focus on studies with stronger experimental design, although performing a randomized trial may raise ethical concerns given the nature and the threat of AL.

In conclusion, our study demonstrates that both SEMS and EVT represent a safe and effective approach for treating AL after upper-GI surgery. Given comparable success rates and lower complications rates, EVT may be preferable to SEMS.

## Figures and Tables

**Table 1:** Clinical and peri-operative characteristics of the study population. SEMS: Self-expanding metal stent; EVT: Endoscopic vacuum therapy; IQR: Interquartile range; BMI: Body mass index.

	Total (n = 45)	SEMS (n = 22)	EVT (n = 23)	*p*-Value
Female, n (%)	12 (26.7)	4 (18.2)	8 (34.7)	0.329
Age, years, median [IQR]	67.0 [20.0]	61.0 [23.0]	70.0 [19.0]	0.103
BMI, kg/m^2^, median [IQR]	24.4 [6.6]	26.6 [9.3]	24.0 [4.6]	0.672
Comorbidities, n (%)				
Cardiovascular	26 (57.8)	14 (63.6)	12 (52.2)	0.295
Respiratory	11 (24.4)	9 (40.9)	2 (8.7)	0.018
Metabolic	16 (35.6)	8 (36.4)	8 (34.7)	>0.999
**Esophageal Cancer, n (%)**	33 (73.3)	20 (90.9)	15 (65.2)	
Histotype				0.864
Adenocarcinoma	17 (52)	11 (55)	6 (46)	
Squamous cell carcinoma	2 (6.1)	1 (5.0)	1 (7.7)	
Other	14 (42)	8 (40)	6 (46)	
Stage				>0.999
I	8 (24)	5 (25)	3 (23)	
II	5 (15)	3 (15)	2 (15)	
III	4 (12)	2 (10)	2 (15)	
IV	16 (48)	10 (50)	6 (46)	
Neoadjuvant therapy, n, (%)	11 (24.4)	5 (25)	6 (40)	0.467
**Gastric Cancer, n (%)**	10 (22.2)	1 (4.5)	9 (39.1)	
Histotype				0.870
Adenocarcinoma	9 (90)	1 (100)	8 (89)	
Squamous cell carcinoma	0 (0)	0 (0)	0 (0)	
Other	1 (10)	0 (0)	1 (11)	
Stage				>0.999
I	0 (0)	0 (0)	0 (0)	
II	1 (13)	0 (0)	1 (14)	
III	2 (25)	0 (0)	2 (29)	
IV	5 (63)	1 (100)	4 (57)	
Neoadjuvant therapy, n, (%)	5 (50)	0 (0)	5 (56)	0.200
Benign Condition, n, (%)	2 (4.4)	1 (4.5)	1 (4.3)	
Surgery, n (%)				0.004
Total Esophagectomies	32 (71.1)	19 (86.4)	13 (56.5)	
Total Gastrectomies	10 (22.2)	2 (9.1)	8 (34.7)	
Other	3 (6.7)	1 (4.5)	2 (8.7)	
Anastomosis				0.009
Thoracic Esophago-Gastric	33 (73.3)	21 (95.4)	13 (56.5)	
Abdominal Esophago-Jejunal	10 (22.2)	1 (4.5)	9 (39.1)	
No anastomosis	2 (4.4)	0	2 (8.7)	

**Table 2:** Clinical characteristics of AL diagnosis and endoscopic features of the study population. SEMS: Self-expanding metal stent; EVT: Endoscopic vacuum therapy; IQR: Interquartile range, AL: Anastomotic leak.

	Total (n = 45)	SEMS (n = 22)	EVT (n = 23)	*p*-Value
Time interval between surgery and diagnosis, days, median [IQR]	6.0 [5.0]	6.0 [5.0]	7.0 [4.8]	0.882
Diagnosis AL, n (%)				0.897
Endoscopy	16 (40)	7 (39)	9 (41)	
CT-scan	24 (60)	11 (61)	13 (59)	
AL size, mm, median [IQR]	6 [7]	5 [5]	8 [9]	0.135
Position of the EVT, n (%)				
Intraluminal	/	/	15 (65.2)	
Extra-luminal	/	/	8 (34.8)	
Time interval changes, days median [IQR]	3.0 [3.5]	21.0 [29]	3.0 [0.0]	<0.001

**Table 3:** Treatment outcomes, adverse events, and mortality of the study population. SEMS: Self-expanding metal stent; EVT: Endoscopic vacuum therapy; IQR: Interquartile range.

	Total (n = 45)	SEMS (n = 22)	EVT (n = 23)	*p*-Value
Technical success, n (%)	45 (100)	22 (100)	23 (100)	>0.999
Clinical success, n (%)	41 (91.1)	19 (86.4)	22 (95.6)	>0.999
Adverse events, n (%)	13 (28.9)	12 (54.5)	1 (4.3)	0.001
Migration	4 (8.9)	4 (18.2)	0 (0)	0.119
Bleeding	0 (0)	0 (0)	0 (0)	>0.999
Stenosis	5 (11.1)	4 (18.2)	1 (4.3)	0.365
Treatment duration, days, median [IQR]	28.0 [21.8]	32.0 [20.5]	22.0 [25.0]	0.029
Number of devices per patient, median [IQR]	2.0 [3.8]	2.0 [1.0]	5.0 [4.0]	<0.001
Additional procedures, n (%)				
Jejunostomy	12 (26.7)	2 (9.1)	10 (43.5)	0.015
SEMS	6 (13.3)	0 (0)	6 (26.1)	0.023
Chest drain	17 (37.8)	14 (63.7)	3 (13.1)	<0.001
Hospital mortality, n (%)	7 (15.6)	5 (22.7)	2 (8.7)	0.225
Overall length of hospital stays, days, median [IQR]	37 [25]	33 [26]	38 [24]	0.864

## Data Availability

The data presented in this study are available on request from the corresponding author due to privacy.

## Supplementary Materials

The following supporting information can be downloaded at: https://www.mdpi.com/article/10.3390/jcm14197075/s1, Figure S1: Schematic comparing SEMS and EVT workflows; Table S1. Multivariable model to assess independent predictors of clinical success; Table S2. Era-adjusted analysis comparing the main outcomes before and after introduction of EVT.
