# Supplementary material for: Stenting Versus Endoscopic Vacuum Therapy for Anastomotic Leakage After Esophago-Gastric Surgery
Source: J Clin Med. 2025 Oct 7;14(19):7075. doi: 10.3390/jcm14197075 (PMC12525109; doi:10.3390/jcm14197075)
Supplement: Supplementary file 1 [file jcm-14-07075-s001.zip › Supplemental Table S1.pdf]

Supplemental Table S1. Multivariable model to assess independent predictors of clinical success. SEMS (self-expanding metal stents); CI: confidence interval

Variable	Odds Ratio	95% CI	P-value
Respiratory comorbidities	0.073	(0.024-1.270)	0.406
Staging	1.160	(0.135-1.460)	0.885
Endoscopic treatment (SEMS)	0.435	(0.001-7.820)	0.778
Intrathoracic anastomosis	9.490	(0.626-3.782)	0.133
Leak size	0.864	(0.588-1.161)	0.366
