# Supplementary material for: Stenting Versus Endoscopic Vacuum Therapy for Anastomotic Leakage After Esophago-Gastric Surgery
Source: J Clin Med. 2025 Oct 7;14(19):7075. doi: 10.3390/jcm14197075 (PMC12525109; doi:10.3390/jcm14197075)
Supplement: Supplementary file 1 [file jcm-14-07075-s001.zip › Supplemental Table S2.pdf]

Supplemental Table S2. Era-adjusted analysis comparing the main outcomes before and after introduction of EVT. EVT: endo-vacuum therapy

	Total (n = 45)	Pre-2018 (n = 18)	≥2018 (n = 27)	p value
Clinical success, n (%)	41 (91)	16 (89)	25 (93)	>0.999
Additional procedures, n (%)				
Jejunostomy	12 (27)	2 (11)	10 (37)	0.086
Chest drain	17 (38)	14 (78)	3 (11)	<0.001
Hospital mortality, n (%)	7 (16)	5 (28)	2 (7.4)	0.098
