# Supplementary figures and images for: Stenting Versus Endoscopic Vacuum Therapy for Anastomotic Leakage After Esophago-Gastric Surgery
Source: J Clin Med. 2025 Oct 7;14(19):7075. doi: 10.3390/jcm14197075 (PMC12525109; doi:10.3390/jcm14197075)

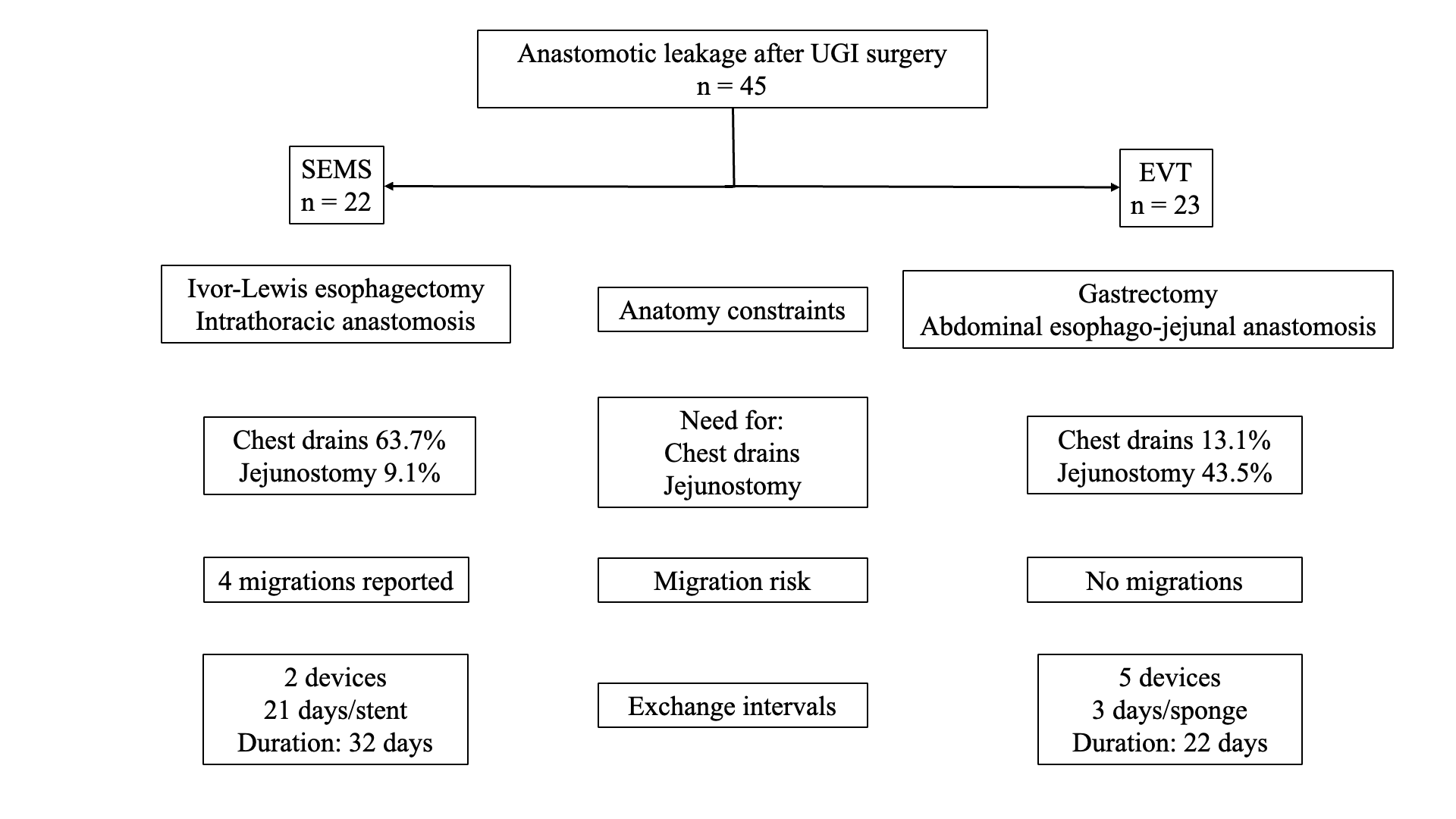

Supplement: Supplementary file 1 [file jcm-14-07075-s001.zip › Supplemental Figure S1.tiff]
